# Preparation of Molecularly Imprinted Cysteine Modified Zinc Sulfide Quantum Dots Based Sensor for Rapid Detection of Dopamine Hydrochloride

**DOI:** 10.3390/molecules28093646

**Published:** 2023-04-22

**Authors:** Xin Zhang, Meng Wang, Yating Zhang, Pan Zhao, Jiamei Cai, Yunjian Yao, Jiarong Liang

**Affiliations:** 1 School of Life Science and Agricultural Engineering, Nanyang Normal University, Nanyang 473061, China; 2 Research Center of Henan Provincial Agricultural Biomass Resource Engineering and Technology, Nanyang 473061, China

**Keywords:** molecular imprinting, quantum dots, dopamine hydrochloride, fluorescent sensor

## Abstract

By combining surface molecular imprinting technology with cysteine-modified ZnS quantum dots, an elegant, molecularly imprinted cysteine-modified Mn^2+^: ZnS QDs (MIP@ZnS QDs) based fluorescence sensor was successfully developed. The constructed fluorescence sensor is based on a molecularly imprinted polymer (MIP) coated on the surface cysteine-modified ZnS quantum dots and used for rapid fluorescence detection of dopamine hydrochloride. The MIP@ZnS quantum dots possess the advantages of rapid response, high sensitivity, and selectivity for the detection of dopamine hydrochloride molecules. Experimental results show that the adsorption equilibrium time of MIP@ZnS QDs for dopamine hydrochloride molecules is 12 min, and it can selectively capture and bind dopamine in the sample with an imprinting factor of 29.5. The fluorescence quenching of MIP@ZnS QDs has a good linear (*R*^2^ = 0.9936) with the concentration of dopamine hydrochloride ranged from 0.01 to 1.0 μM, and the limit of detection is 3.6 nM. In addition, The MIP@ZnS QDs demonstrate good recyclability and stability and are successfully employed for detection of dopamine hydrochloride in urine samples with recoveries was 95.2% to 103.8%. The proposed MIP@ZnS QDs based fluorescent sensor provides a promising approach for food safety detection and drug analysis.

## 1. Introduction

Dopamine hydrochloride (3-hydroxytyramine hydrochloride, DA-HCL) is considered a central neurotransmitter which plays an important role in cardiovascular, central nervous system, renal function regulation, and so on [1,2]. As a β-stimulant, dopamine hydrochloride has the function of accelerating metabolism and inhibiting lipogenesis. It is illegally added to foodborne animal feed as a “clenbuterol” drug, causing great concern for food safety supervision [3,4]. Such substances can accumulate in the human body through contaminated food, causing acute poisoning symptoms such as palpitation, arrhythmia, nausea and vomiting, inducing Parkinson’s disease, hypertension and gastrointestinal dysfunction [5,6,7]. At present, the detection methods of dopamine hydrochloride include high performance liquid chromatography (HPLC), HPLC-mass spectrometry, solid phase extraction-HPLC, enzyme-linked immunosorbent assay, electrochemistry, chemical/biological sensing, and so on [8,9,10,11,12,13]. However, many of these detection techniques require complex sample pretreatment processes or high-precision instruments, requiring professional and technical personnel to operate, and long detection cycles. Therefore, it is important to develop a low-cost, rapid, convenient, and highly selective method for detecting dopamine hydrochloride.

Quantum dots (QDs) are nanoscale semiconductors with quantum confinement of electrons and holes [14,15]. Quantum dots are highly valuable in biological analysis and sensing due to their unique characteristics, such as quantum size effect, size-dependent emission spectrum, specific surface area, good fluorescence stability, and high luminescence efficiency [16,17,18]. To date, there has been significant work to develop quantum dots-based sensors with unique physical and optical properties. Quantum dots-based sensors can rapidly and sensitively detect target analytes by the fluorescence quenching of QDs [19,20]. However, the selectivity to target analytes is a challenge in the design of QD-based sensors. Molecular imprinting is a simple technology to construct polymers that can specifically recognition of target molecules by copolymerizing functional monomers with template molecule [21]. The template molecules were removed by elution, and the complementary binding sites were formed on the molecularly imprinted polymer (MIP). MIP possesses the merits of strong mechanical stability, low-cost preparation procedure and specificity to the target molecule [22,23,24]. For the fabrication of the traditional MIP, template molecules will be embedded into the polymer, which is not conducive to elution and mass transfer and affects the adsorption and specificity of the target molecule, limiting the application of this of this technology [25,26]. In contrast, surface molecular imprinting technique (SMIT) is a new analytical method with great potential. Surface molecular imprinting is the fabrication of a “core-shell” structure polymers by coated MIPs on the surface of solid substrate nanomaterials [27,28,29]. Thus, the imprinted binding site is distributed on the surface of the molecularly imprinted polymer or the outer layer of the matrix material so that the template molecule is more easily closer to the binding site. MIPs prepared by SMIT method have formed more binding sites during the imprinting process, which facilitated rebinding template molecules and high adsorption capacity. It is an ideal approach to solve the problems of slow mass transfer, difficult to elute template molecules, and low imprinting efficiency of the traditional imprinted polymer [30,31,32].

In this work, cysteine was chosen to modify the surface of manganese doped zinc sulfide QDs to enhance the fluorescence stability, dispersion ability in the aqueous phase, and biocompatibility of Mn^2+^: ZnS QDs. Moreover, cysteine modification can provide carboxyl group for QD, which facilitates the binding of quantum dots with template molecules. Using methacrylic acid as function monomer and ethylene glycol dimethyl acrylate as crosslinkers, and cysteine modified Mn^2+^: ZnS quantum dots as substrate, the dopamine molecularly imprinted cysteine modified Mn^2+^: ZnS quantum dots (MIP@ZnS QDs) were fabricated via surface molecular imprinting. The constructed MIP@ZnS QDs integrates the fluorescent characteristic of ZnS QDs and the selective molecular recognition capability of MIPs. The MIP@ZnS QDs has good physical/chemical stability, easy elution/adsorption, fast mass transfer, and high selectivity for target molecules [33,34,35,36,37]. The MIP layer on the QDs can specifically bind and capture target molecules, which will result in fluorescence quenching of quantum dots due to the non-radiative transition between the bound target molecules and quantum dots [38,39,40]. Therefore, the molecular recognition process is transformed into photoelectric signal analysis, and the quenching of MIP@ZnS quantum dots is proportional to the amount of dopamine hydrochloride molecules bound. Thus, the prepared MIP@ZnS QDs can be applied to the high sensitivity and selectivity analysis of dopamine hydrochloride. This work also provides a new method for the analysis of harmful chemical substances in environment and food safety.

## 2. Results and Discussion

### 2.1. Preparation of MIPs@ZnS QDs

**Figure 1:**
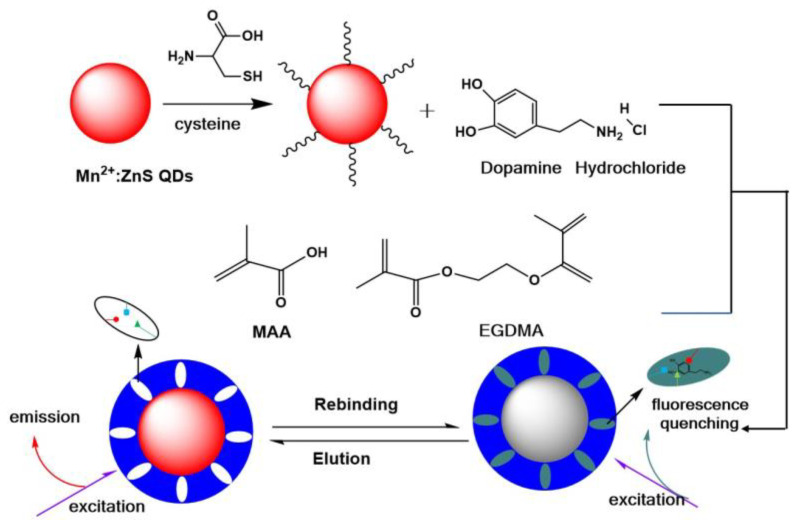
Schematic of preparation of dopamine hydrochloride molecularly imprinted cysteine-modified Mn^2+^: ZnS QDs-based fluorescent sensor. dopamine hydrochloride: 2.0 mg; Mn^2+^: ZnS QDs: 100 mg.

The fabrication process of the dopamine hydrochloride fluorescent sensor is depicted in Figure 1. First, Mn^2+^: ZnS QDs were used as the signal transduction element and solid substrate nanomaterials. The ZnS quantum dots have narrow emission bands, and the Mn^2+^ doped in ZnS QDs can further improve the fluorescence quantum yield and photochemical stability of quantum dots [41]. In addition, Mn^2+^ doped ZnS QDs has water dispersibility and low cytotoxicity. The cysteine was modified on Mn^2+^: ZnS QDs to enhance the fluorescence stability and dispersion in aqueous. Moreover, cysteine modification can provide carboxylic acid group (-COOH) on the surface of QDs, which promotes the binding of monomers with template molecules and makes it easier to form recognition cavies. The cysteine-modified Mn^2+^: ZnS QDs can also be used as assistant monomers contributing to form imprinting cavies. Second, methacrylic acid was chosen as a functional monomer and EGDMA as a crosslinker; the dopamine hydrochloride molecules were imprinted on cysteine-modified Mn^2+^: ZnS QDs though sol-gel reaction. The constructed MIP@ZnS QDs has a core-shell structure. The MIP layer on the surface of MIP@ZnS QDs can not only selectively capture the template molecule (dopamine hydrochloride), but also prevent the interaction between other interfering substances and the quantum dot. The MIP shell on the QDs can effectively reduce the influence caused by the interfering substances. Finally, the MIP@ZnS QDs were eluted by acetic acid-methanol solvent to remove imprinted template molecules until the fluorescence intensity of MIP@ZnS QDs was not changed and similar to that of NIP. The formed imprinting sites was complementary to dopamine hydrochloride molecules in shape, size and chemical structure. The developed molecularly imprinted quantum dot can be specifically combined with dopamine hydrochloride molecules through a molecularly imprinted layer, and the fluorescence of the quantum dot is quenched because of the energy transfer effect of electrons between the quantum dot and dopamine hydrochloride molecules.

### 2.2. Characterization

**Figure 2:**
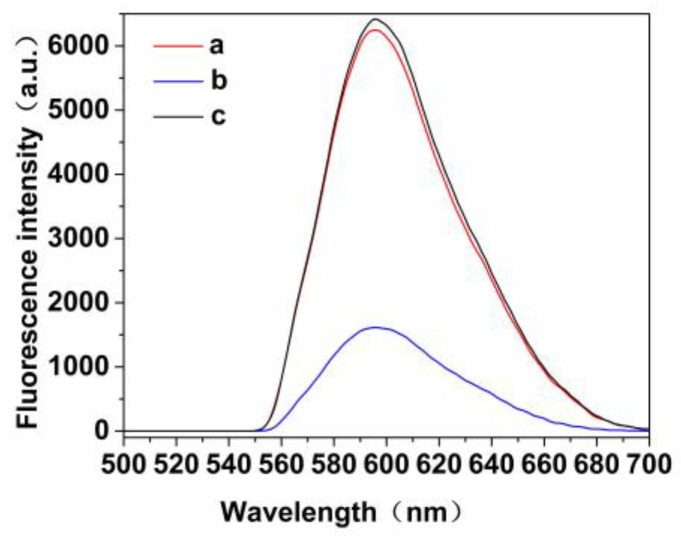
Fluorescence spectrum of (**a**) MIP@ZnS QDs elution of template molecule; (**b**) MIP@QDs before elution of template molecule, and (**c**) NIP@ZnS QDs.

Fluorescence spectra indicated that the maximum excitation and emission peaks of MIP@ZnS QDs are located at 320 nm and 596 nm, respectively. As shown in Figure 2, the prepared MIP@ZnS QDs has symmetrical emission peaks and orange fluorescence emission. The full width at half maximum (FWHM) of MIP@ZnS QDs is 40 nm, and no visible defect peaks were found. In addition, before the elution of dopamine hydrochloride molecules, the intensity of MIP@ZnS QDs was only 25.2% of that of NIP@ZnS QDs. The fluorescence quenching is due to the amount of template molecule binding on the MIP@ZnS QDs, which leads to a charge transfer interaction between the QDs and the dopamine hydrochloride molecule. After the elution, the fluorescence emission of MIP@ZnS QDs recovered significantly, and the fluorescence intensity was 97.3% of NIPs.

**Figure 3:**
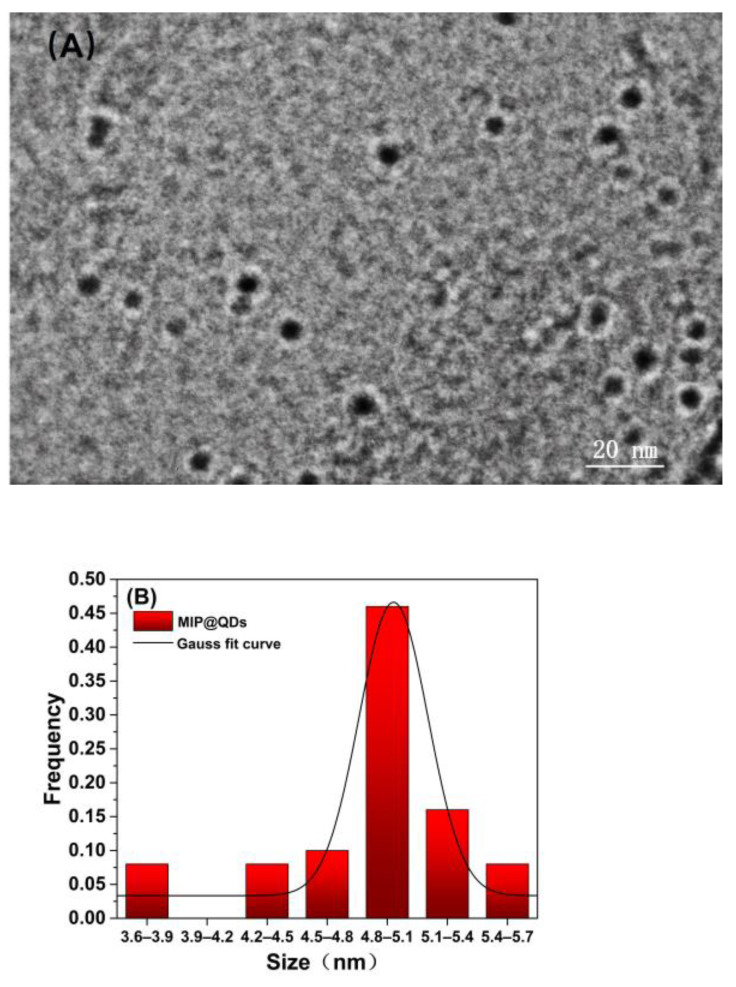
(**A**) TEM image and (**B**) particle size distribution of prepared MIP@QDs.

The structure and morphology of the MIP@ZnS QDs were characterized using JF-2100 transmission electron microscope (TEM). Figure 3 revealed that the MIP@ZnS QDs possessed spherical shape, and particle size distribution of prepared MIP@ZnS QDs is about 4.9 ± 0.53 nm.

### 2.3. Effect of the Monomer on MIP@ZnS QDs

**Figure 4:**
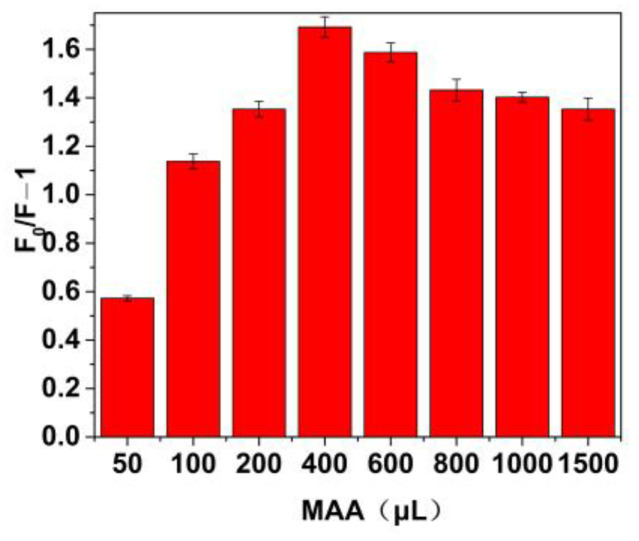
Effect of monomer (MAA) amount on the fluorescence quenching degree of MIP@ZnS QDs.

The amount of functional monomer (MAA) is major influencing factor in the molecular imprinting process. The changes of fluorescence quenching (Δ*F*) of MIP@ZnS QDs under the different amount of MAA are present in Figure 4. As the amount of MAA increases (0~400 μL), the fluorescence quenching of MIP@ZnS quantum dots gradually enhances. When the dosage of MAA monomer is 400 μL, the highest quenching effect of dopamine hydrochloride on MIP@ZnS quantum dots is achieved. Experimental results indicate that a low dosage of MAA is insufficient to form an MIP layer on the cysteine-modified ZnS QDs, resulting in inadequate binding sites for dopamine in molecular imprinting. Excessive use of monomers can increase the thickness of the MIP layer, which is detrimental to the entry of dopamine hydrochloride molecules into the binding site and affects the mass transfer efficiency. Additionally, an excess of crosslinker agents during preparation can also hinder the binding of target molecules on MIP@ZnS quantum dots.

### 2.4. Effect of pH on MIP@ZnS QDs

**Figure 5:**
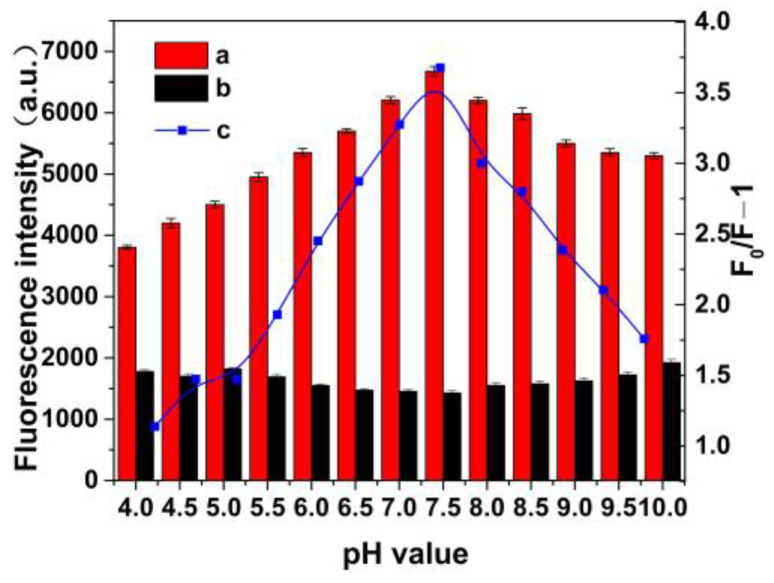
The fluorescence intensity of (**a**) MIP@ZnS QDs and (**b**) MIP@ZnS QDs bound with dopamine hydrochloride molecule at different pH values; (**c**) the change of fluorescence quenching of MIP@ZnS QDs at different pH values. Dopamine hydrochloride: 0.5 μM; room temperature.

Figure 5 shows that the fluorescence intensity of MIP@ZnS quantum dots increases with the pH values in the range of 4.0 to 7.0. The instability of the structure of MIP@ZnS quantum dots in acidic environments leads to a decrease in their fluorescence performance. The experiment shows that dopamine hydrochloride molecules are more easily bound with MIP@ZnS quantum dots in weak alkaline environments, with significant fluorescence quenching efficiency. When the solution pH value is 7.5, the fluorescence quenching of MIP@ZnS quantum dots reaches the maximum value. Therefore, the pH value of 7.5 set in the experiment is the optimal pH value for detecting hydrochloride dopamine with MIP@ZnS quantum dots.

### 2.5. Response Time

**Figure 6:**
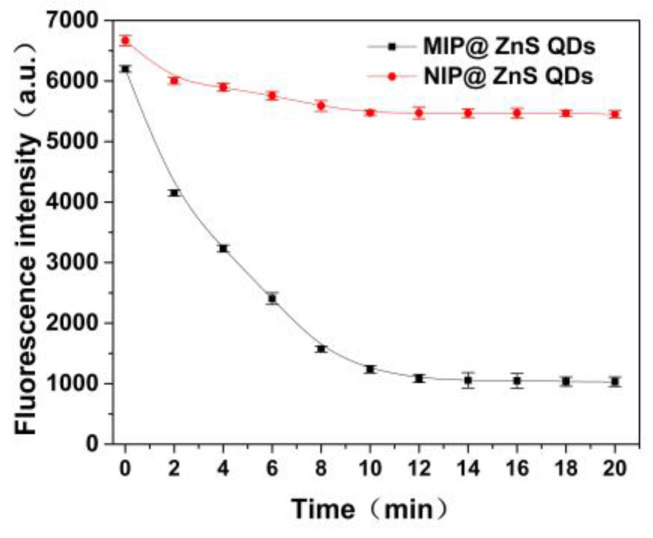
Binding kinetic curve of MIP@ZnS QDs and NIP@ZnS QDs. Dopamine hydrochloride: 0.5 μM; room temperature.

As shown in Figure 6, the degree of fluorescence quenching (Δ*F*) of dopamine hydrochloride molecules on MIP@QDs increases with the increase of incubation time (0–12 min). Dopamine hydrochloride molecules have a significant fluorescence quenching effect on MIP@ZnS quantum dots. The fluorescence of MIP@ZnS QDs tended to stabilize after incubation for 12 min, indicating that the adsorption and binding of dopamine hydrochloride molecules on MIP@ZnS QDs reached dynamic equilibrium. As the control group, the NIP@ ZnS QDs showed slight fluorescence change in the samples reaching equilibrium at around 10 min. It is due to the lack of molecular imprinting sites for dopamine hydrochloride molecules on NIP@ZnS QDs; the binding of dopamine hydrochloride molecules is mainly through physical adsorption [42,43]. The experimental results suggest that the response time of the prepared MIP@ZnS quantum dots sensor in the sample solution is 12 min.

### 2.6. Determination of Dopamine Hydrochloride by MIP@ZnS QDs

In this experiment, the principal detection method is based on fluorescence quenching of MIP@ZnS QDs by dopamine molecules. The prepared MIP@ZnS QDs possessed orange fluorescence emission was generated by photoexcitation of the host ZnS nanocrystal recombined by the lower-lying states of the dopped Mn^2+^ ion [38]. The MIP@ZnS QDs could selectively capture target molecules (DA) through complementary imprinting sites on the molecularly imprinted polymers of Mn^2+^: ZnS QDs. This specific binding process introduces electron transfer of Mn^2+^: ZnS QDs to the target molecule (DA), which leads to fluorescence quenching of MIP@ZnS QDs [39]. Moreover, the quenching degree of MIP@ZnS QDs was positively correlated with the concentration of target molecules in the samples.

**Figure 7:**
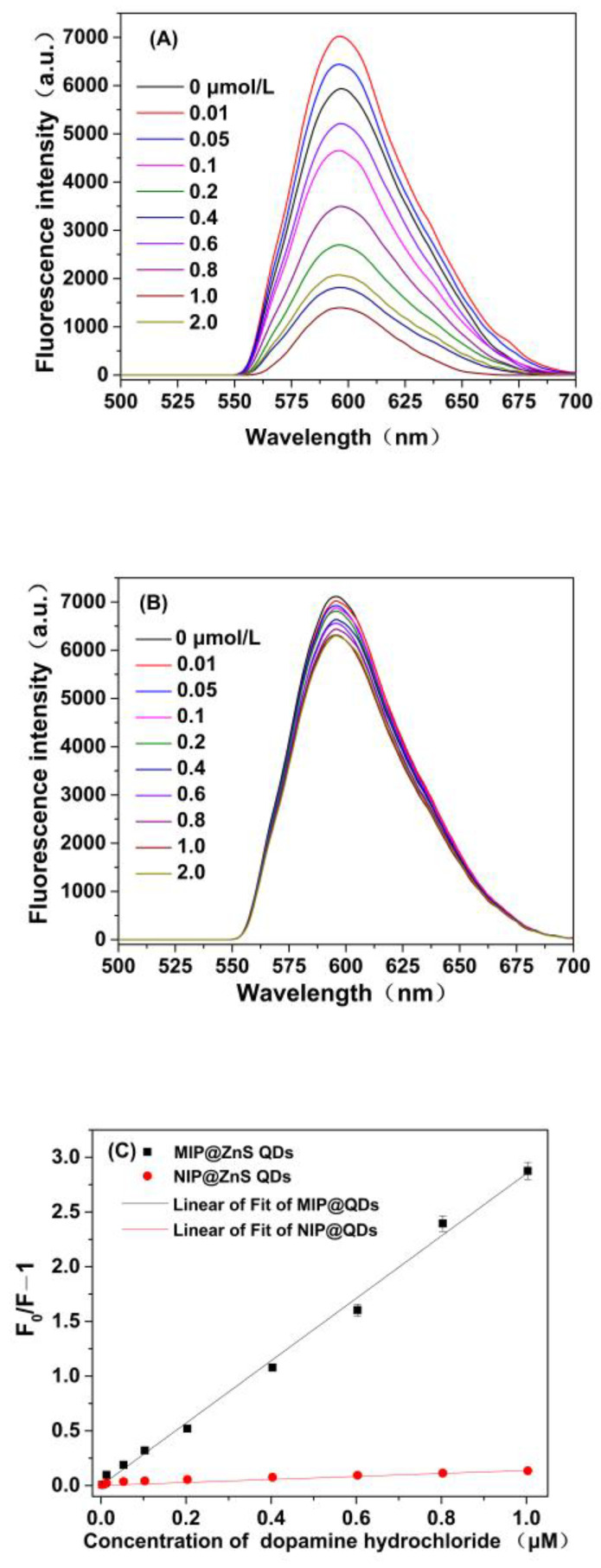
Fluorescence emission spectra of (**A**) MIP@ZnS QDs and (**B**) NIP@ ZnS QDs at different concentrations of dopamine hydrochloride; (**C**) Fitting curves of fluorescence quenching values (*F*_0_*/F* − 1) against the concentration of dopamine hydrochloride; Experimental conditions: room temperature, concentrations of dopamine hydrochloride: 0.01~1.0 μM.

Figure 7 depicts the fluorescence emission spectra of the prepared MIP@ZnS and NIP@ZnS quantum dots under different concentrations of dopamine hydrochloride. With increased concentrations of hydrochloric acid dopamine in the sample, the fluorescence spectra of MIP@ZnS and NIP@ZnS QDs presented significant changes. Compared with NIP@ ZnS QDs, MIP@ZnS quantum dots exhibit stronger fluorescence quenching in their fluorescence spectra, with a fluorescence quenching degree (*F*_0_*/F* − 1) much higher than that of NIP@ ZnS quantum dots. MIP@ZnS quantum dots possess binding sites that complement the shape, size, and functional groups of dopamine hydrochloride molecules through non-covalent interactions such as hydrogen bonding and van der Waals forces [21,44]. Due to the presence of the imprinting sites, more dopamine hydrochloride molecules specifically bound to MIP@ZnS QDs and resulted in significant fluorescence quenching. In contrast, dopamine hydrochloride molecules mainly bound to NIP@QDs through non-specific adsorption, limiting the amount of dopamine molecules adsorption on the NIPs. Therefore, NIP@ZnS QDs displayed lower fluorescence quenching than that of MIPs. Figure 7A showed that the fluorescence quenching of MIP@ZnS QDs is proportion to the concentration of dopamine hydrochloride in the samples. Curve fitting (Figure 7C) indicates that there is a good linear relationship (*R*^2^= 0.9936) between *F*_0_*/F* − 1 and concentration of dopamine hydrochloride (*Q*) in the range of 0.01~1.0 μM. The Stern–Volmer equation is *F*_0_*/F* = 2.8445 Q – 0.00373. The limit of detection is calculated by 3*δ*/*S* (IUPAC criteria), *δ* is the standard deviation of the blank signal (*n* = 20), and *S* is the slope of the linear calibration plot. The corresponding detection limit of MIP@ZnS QDs for dopamine is 3.6 nM.

### 2.7. Selective Experimental Methods

**Figure 8:**
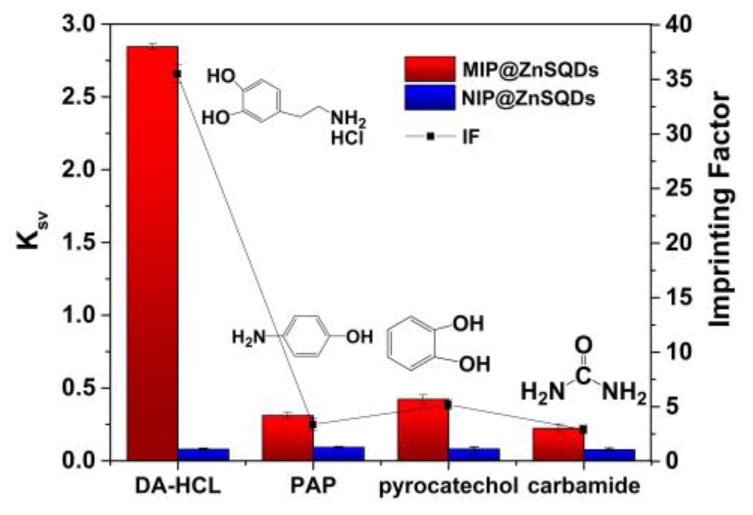
Quenching constants of MIP@ZnS QDs, NIP@ ZnS QDs and imprinting factors (IF) for dopamine hydrochloride (DA-HCl), *p*-aminophenol (PAP), pyrocatechol and carbamide.

In selective experiments, dopamine hydrochloride significantly exhibited fluorescence quenching constants on the MIP@ZnS QDs compared with other molecules (Figure 8). During the molecular imprinting process, a series of binding sites were formed on the cysteine-modified Mn^2+^: ZnS QDs, which complement the DA molecule in shape, structure, size and functional groups. Due to the presence of these molecular imprinting binding sites, MIP@ZnS QDs is more likely to bind with DA molecules. Therefore, a large amount of dopamine hydrochloride molecules were specifically bound to MIP@ZnS QDs, which results in significant fluorescence quenching. However, as reference compounds, *p*-aminophenol, pyrocatechol and carbamide molecules are different in shape, size, and functional groups from dopamine hydrochloride molecules. These structural analog molecules are only bound to MIP through non-specific effects such as physical adsorption and cannot match the molecularly imprinted cavies on MIP@ZnS QDs. Thus, their adsorption capacity and fluorescence quenching degree were lower on MIP@ZnS QDs. As a control, NIP@QDs showed similar fluorescence quenching degree because there are no molecular imprinting sites formed during the preparation process. The imprinting factor of MIP@ZnS QDs for dopamine hydrochloride is 29.5, which is much higher than that of p-aminophenol and pyrocatechol and carbamide (3.6, 5.9, and 2.7, respectively). The experimental results indicated that MIP@ZnS QDs have good selectivity for dopamine hydrochloride. The selectivity of MIP@ZnS QDs to DA molecules is mainly due to the non-covalent interactions (hydrogen bonding, van der Waals forces, etc.) formed between functional monomers, crosslinkers and template molecules during the preparation process. The selectivity of MIP@ZnS quantum dots to DA molecules is mainly due to the non-covalent interaction (hydrogen bond, van der Waals force, etc.) between functional monomers and template molecules and the formation of complementary molecular imprinting cavities in the molecularly imprinting process. The method was compared with other reported dopamine hydrochloride detection techniques (Table 1). Compared with the reported detection methods based on QDs and other novel nanomaterials (COF, GQD, graphene), MIP@ZnS QDs based fluorescence sensors exhibit satisfactory linear range, detection limit, and good recovery rates for the detection of dopamine hydrochloride. In addition, the developed methods have the advantages of simple and convenient operation, low equipment requirement without any complex sample treatment process, and have better selectivity.

### 2.8. Application

In this study, real samples of bovine urine, sheep urine and human urine were used to evaluate the practical application performance of the MIP@ZnS QDs based sensor. As shown in Table 2, dopamine hydrochloride was not detected in any of the three urine samples. In order to further verify the accuracy of MIP@ZnS quantum dot sensor in detecting dopamine hydrochloride, a standard addition method was used to conduct recovery experiments on dopamine hydrochloride in the samples. The recoveries of dopamine hydrochloride in different samples ranged from 95.2% to 103.8% with RSD of 3.75% to 5.73%. The results show that the developed molecularly imprinted quantum dots sensor exhibited satisfactory reliability and accuracy in the detection of dopamine hydrochloride in real samples.

### 2.9. Recyclability and Stability

**Figure 9:**
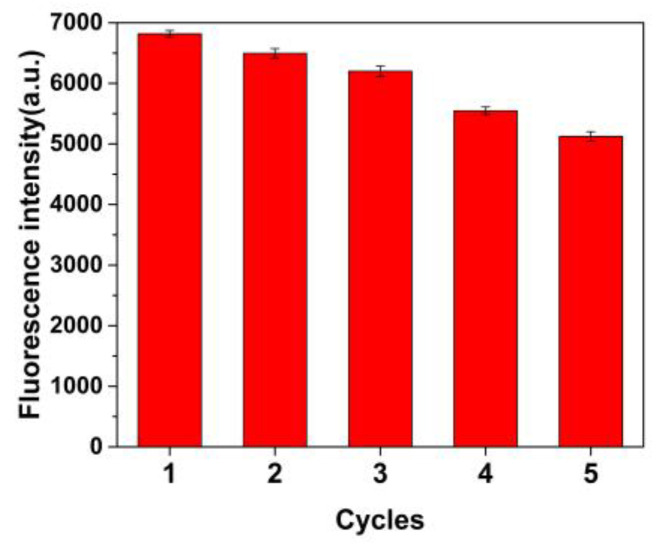
Recyclability test of MIP@ZnS QDs.

**Figure 10:**
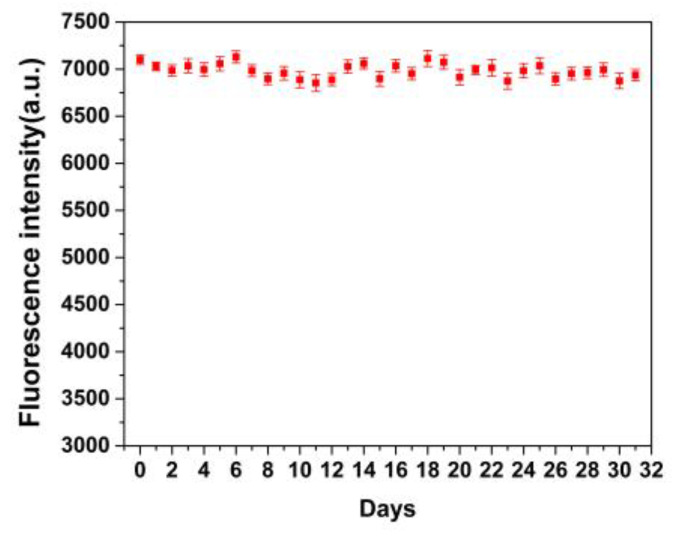
Fluorescence stability of MIP@ZnS QDs.

As shown in Figure 9, the regeneration cycle (adsorption/elution/re-adsorption) was carried out to investigate the recyclability of MIP @ZnS QDs. After four cycles, the fluorescence of MIP@ZnS QDs significantly decreased. Compared with the initial value of the sensor, the fluorescence intensity has decreased by about 20%. Furthermore, the fluorescence stability of the MIP@ZnS QDs was evaluated and depicted in Figure 10. After being stored in the dark for 31 days; the fluorescence intensity of the MIP@ZnS QDs has no significant changes compared to the initial, indicating good stability of the MIP@ZnS QDs. These experimental results indicate that the designed MIP@ZnS QDs-based sensor has good recyclability and stability and has high practical application value as a fluorescence sensor.

## 3. Materials and Methods

### 3.1. Instruments and Reagents

Dopamine hydrochloride, ZnSO_4_·7H_2_O, MnCl_2_·4H_2_O, Na_2_S·9H_2_O, *p*-aminophenol (PAP), and carbamide were obtained from Macklin Biochemical Co. Ltd. (Shanghai, China). Pyrocatechol, cysteine, methacrylic acid (MAA), ethylene glycol dimethyl acrylate (EGDMA) was purchased from J&K Scientific (Beijing, China). All the reagents are of analytical grade.

Fluorescence spectrometer (Techcomp FL-970, Shanghai, China). Ultraviolet spectrophotometer (Specord 210 plus, Jena, Germany). High-resolution transmission microscope (JEM-2100F, JEOL, Tokyo, Japan)

### 3.2. Preparation of Cysteine Modified Mn^2+^: ZnS QDs

Cysteine modified Mn^2+^: ZnS QDs were prepared by chemical precipitation method with some modifications [49,50]. In brief, 1.80 g ZnSO_4_·7H_2_O, 0.10 g MnCl_2_ 4H_2_O, were ultrasonically dispersed into 20 mL ultrapure water, and stirred for 1.0 h under nitrogen protection at 25 °C. Then, 5.0 mL of Na_2_S·4H_2_O solution was dropwise added. After stirring another 10 h, 10 mg of cysteine was added and continued to be stirred for 12 h in the dark. Finally, the product was washed and separated by centrifuging, and dried under vacuum.

### 3.3. Preparation of MIP@ZnS QDs

The dopamine molecularly imprinted layer was constructed using surface imprinting technology onto the surface of cysteine modified Mn^2+^: ZnS QDs. First, 2.0 mg dopamine hydrochloride is dissolved in 10 mL of methanol, 400 μL of MAA is added to the flask and stirred for 10 min. Second, 100 μL of EGDMA and 100 mg of cysteine modified Mn^2+^: ZnS QDs were added. Third, AIBN (15 mg) was added, and UV (360 nm) initiated polymerization at 50 °C in the dark for 20 h. Finally, the prepared product was collected by centrifugation and washed with methyl alcohol. The non-imprinted ZnS QDs (NIP@ZnS QDs) were synthesized by the same procedure without dopamine hydrochloride molecules. The obtained product was eluted by methanol and acetic acid (9:1, *v/v*) solution to remove dopamine hydrochloride molecules until no dopamine hydrochloride molecules were detected in the eluent. The eluted product is washed to be neutral by methanol and is freeze-dried in vacuum for further use.

### 3.4. Effect of pH on MIP@ZnS QDs

20 mg of MIP@ZnS QDs were ultrasonically dispersed in dopamine hydrochloride solutions with different pH values. The dopamine hydrochloride stock solution was added and diluted with buffer solution. The concentration of dopamine hydrochloride in the sample was 0.5 μM. Using the solution without dopamine hydrochloride as a control, the change of the fluorescence intensity of the samples were analyzed by fluorescence spectrofluorometer.

### 3.5. Determination of Dopamine Hydrochloride Using MIP@ZnS QDs

To carry out the rebinding experiments, 19 mg of dopamine hydrochloride standard solution is dissolved in 100 mL of methanol as the stock solution. 20 mg of MIP@ZnS QDs or NIP@ZnS QDs are dissolved in 100 mL of pH 7.5 buffer solution, and 5 mL of the polymer solution is taken into a 10-mL calibrated test tube after ultrasonic dispersion. Then, a certain amount of dopamine hydrochloride standard solution is sequentially added and diluted to 10 mL. The concentration of dopamine hydrochloride ranging from 1.0 × 10^−8^ to 1.0 × 10^−6^ mol/L. After incubating for 12 min, the fluorescence emission spectra with different dopamine hydrochloride concentrations was recorded. The fluorescence quenching of the MIP@ZnS QDs in the system is calculated by the Stern-Volmer equation [51]:

(1) F0F−1=Ksv[Q]

*F*_0_ is the initial fluorescence intensity in the absence of the dopamine hydrochloride molecule; *F* is the fluorescence intensity with dopamine hydrochloride; *K_sv_* is the quenching constant of the equation; *Q* is the concentration of the dopamine hydrochloride. The degree of quenching of the prepared MIP@QDs by dopamine hydrochloride molecules is represented by ΔF (Δ*F* = *F*_0_/*F* − 1).

### 3.6. Selective Experiment

*p*-Aminophenol, pyrocatechol, and carbamide were used as structural analogues to conduct the selectivity experiments. Dopamine hydrochloride, *p*-aminophenol, pyrocatechol and carbamide stock solution were prepared. After ultrasonic dispersion, 5 mL of the MIP@ZnS QDs and NIP@ZnS QDs dispersed solution is taken into a 10-mL calibrated test tube. Then, a certain amount of the structural analogues standard solution was added and diluted to 1.0 × 10^−8^~1.0 × 10^−6^ mol/L. After incubation that lasted 12 min, the changes in fluorescence intensity of samples were measured. The imprinting factor (IF) of MIP@ZnS QDs for each compound was calculated as follows:

(2) $$IF=K_{SV,MIP}/K_{SV,NIP}$$

*K_SV,MIP_* and *K_SV,NIP_* are the quenching constants of MIP@ZnS QDs and NIP@ZnS QDs in different substance solutions, respectively.

### 3.7. Real Sample Analysis

Practical application is one of the important indicators to evaluate the prepared sensor. The samples were collected from the urine of cattle, sheep and healthy volunteers. All samples were filtered with 0.45 μm filter and stored in a 4 °C refrigerator. The prepared MIP@ZnS QDs were dispersed in 10 mL sample solution. After incubation, the changes of fluorescence intensity in the sample solutions were measured and the concentration of dopamine hydrochloride in the samples was calculated. To further verify the reliability of the method, a recovery test was conducted using standard addition method.

## 4. Conclusions

In this study, a molecular imprinted fluorescence sensor for dopamine hydrochloride was successfully designed by combining water-soluble ZnS QDs with surface molecular imprinting technology. After binding with dopamine hydrochloride molecules, MIP@ZnS QDs undergo significant quenching and the quenching is proportional to the concentration of the dopamine hydrochloride molecules. The constructed MIP@ZnS QDs sensor presents high selectivity towards dopamine hydrochloride and has been successfully used for real sample determination. The developed MIP@ZnS QDs based fluorescence sensor has advantages such as simple operation, fast response, low cost, and high stability and selectivity. It has great potential in food safety, biosensing, environmental protection, and other fields.

## Figures and Tables

**Table 1:** Comparison of analysis results of dopamine hydrochloride by this method and other reported methods analyte.

Detection Technique	Linearity Range (μM)	LOD (nM)	Recoveries (%)	References
COF/Pt/MWCNT-COOH	2.0–500	670	91.7–110.9	[12]
SPE-HPLC	1.0–15.0	170	98.3–101.1	[8]
GQDs	0–60.0	8.0	92.6–106.8	[45]
Graphene	5.0–2000	200	/	[46]
Adenosine capped QDs	0.1–20.0	29.3	94.8–103.4%	[47]
l-cysteine-ZnS: Mn QDs	0.15–3.0	7.80	80–93%	[48]
MIP-QDs	0.026–1.58	10.50	71.74–108.63	[13]
MIP@ZnS QDs	0.01–1.0	3.60	95.2–103.8	This work

COF: covalent organic frameworks; SPE: solid phase extraction; HPLC: high performance liquid chromatography; GQDs: graphene quantum dots; MIP: molecularly imprinted polymer; QDs: quantum dots.

**Table 2:** Detection of dopamine hydrochloride in actual samples by MIP@QDs.

Samples	Added (μM)	Detection of DA-HCl	Recovery (%)	RSD (%, *n* = 3)
bovine urine	0.1	0.0952	95.2	4.23
	0.5	0.4930	98.6	5.18
sheep urine	0.1	0.1035	103.5	3.75
	0.5	0.5191	103.8	5.73
human urine	0.1	0.1022	102.2	3.86
	0.5	0.4921	98.4	4.84

## Data Availability

All the data generated by this research is included in the article.
